# Sinonasal Microbiome Sampling: A Comparison of Techniques

**DOI:** 10.1371/journal.pone.0123216

**Published:** 2015-04-14

**Authors:** Ahmed Bassiouni, Edward John Cleland, Alkis James Psaltis, Sarah Vreugde, Peter-John Wormald

**Affiliations:** Department of Surgery—Otorhinolaryngology, Head & Neck Surgery, University of Adelaide, Adelaide, Australia; Hospital of the University of Pennsylvania, UNITED STATES

## Abstract

**Background:**

The role of the sino-nasal microbiome in CRS remains unclear. We hypothesized that the bacteria within mucosal-associated biofilms may be different from the more superficial-lying, free-floating bacteria in the sinuses and that this may impact on the microbiome results obtained. This study investigates whether there is a significant difference in the microbiota of a sinonasal mucosal tissue sample versus a swab sample.

**Methods:**

Cross-sectional study with paired design. Mucosal biopsy and swab samples were obtained intra-operatively from the ethmoid sinuses of 6 patients with CRS. Extracted DNA was sequenced on a Roche-454 sequencer using 16S-rRNA gene targeted primers. Data were analyzed using QIIME 1.8 software package.

**Results:**

At a maximum subsampling depth of 1,100 reads, the mean observed species richness was 33.3 species (30.6 for swab, versus 36 for mucosa; p > 0.05). There was no significant difference in phylogenetic and non-phylogenetic alpha diversity metrics (Faith’s PD_Whole_Tree and Shannon’s index) between the two sampling methods (p > 0.05). The type of sample also had no significant effect on phylogenetic and non-phylogenetic beta diversity metrics (Unifrac and Bray-Curtis; p > 0.05).

**Conclusion:**

We observed no significant difference between the microbiota of mucosal tissue and swab samples. This suggests that less invasive swab samples are representative of the sinonasal mucosa microbiome and can be used for future sinonasal microbiome studies.

## Introduction

Recent advances in high-throughput DNA sequencing technology have revolutionized bacterial detection techniques. Contrary to traditional isolation methods, which are prone to biases inherent in the varying abilities of bacteria to grow in culture, sequencing allows direct examination of the DNA content in a sample. Based on these DNA readings, we are thus able to infer the bacteria contained within. This molecular method is very sensitive and is able to provide a more complete picture of all the microbes living in a certain environment, what is termed the microbiome. Recently there has been increased interest in sinonasal microbiome research, and how changes in the microbiome could relate to sinus conditions such as chronic rhinosinusitis (CRS). Recent findings point towards reduced microbial diversity in CRS patients, when compared with healthy controls.[1,2] To the best of our knowledge, almost all 16S rRNA sinonasal microbiome studies to date have relied upon sampling via swabs[3–5] or via sinus lavage[2,6], with no studies examining the microbiome of mucosal biopsies. Recent parallel research does suggest that bacteria existing within the sinus mucosa itself,[7,8] or adherent to the mucosa in the form of a bacterial biofilm, is associated with the disease state of CRS. [9] Numerous studies have demonstrated biofilms to be associated with an inflammatory response in the underlying mucosa,[10–13] and clinical studies suggested that these bacterial forms may predispose to more severe sinus disease and worse post-operative outcomes.[14,15] Traditionally, particular bacterial species like *Staphylococcus aureus* and *Pseudomonas aeruginosa* have been associated with these worse outcomes.[16–19]

Knowing this, we hypothesize that mucosal swabs may miss capturing these resistant bacterial forms leading to a mis-representation of the true sinonasal microbiome. To investigate this we compare the microbiome results of swab samples and mucosal tissue biopsy samples, taken from the same patients.

## Methods

### Ethics statement

All participants underwent informed consent, with written consent being obtained prior to enrollment. This study was approved by the local institutional review board (The Queen Elizabeth Hospital Human Research Ethics Committee), application number: 2011008 (amended).

### Study design

This observational cross-sectional study uses a paired design to compare, in the same patients, two different types of sampling for investigating the sinonasal microbiome—the first type of sample is a swab, while the second type of sample is a mucosal tissue biopsy. Both types of samples were obtained intra-operatively from the ethmoid sinuses of patients with CRS.

### Study participants

Six CRS patients attending the tertiary Rhinology practice of the senior author (P.J.W.) and The Queen Elizabeth Hospital (Adelaide, Australia) for primary or revision endoscopic sinus surgery (ESS) were included in this study. Patients who administered antibiotics or antifungals in the previous week prior to surgery were excluded from the study. Surgery received was “full-house” ESS (which includes middle meatal antrostomies, fronto-ethmoidectomies and sphenoidectomies). Some patients also had a Draf-III/frontal drillout procedure and/or canine fossa trephination (CFT) for severe frontal or maxillary disease.

### Sample collection

All samples were collected intra-operatively. Endoscopically-guided swabs were taken from the anterior ethmoid sinus. We used flocked swabs (Copan Italia S.p.A., Brescia, Italy) to maximize bacterial yield. After swabbing, mucosal tissue biopsies were taken from the corresponding region in the anterior ethmoid sinuses using a standard-sized Blakesley forceps (Size 3, cup size 5×15mm; Karl Storz, Tuttlingen, Germany). Samples were always taken from this region, irrespective of the presence or absence of pus. To avoid inadvertent contamination, any swabs that could have come into contact with the nasal vestibule during sampling were discarded. The swab head was then immediately separated into a sterile container, placed on ice, and then transported to the laboratory for storage at -80°c.

### DNA extraction

Swab heads were thawed, cut into small pieces and then placed in 180 μl of enzymatic lysis buffer (Qiagen, CA, USA) overnight at room temperature. Bead homogenization was then performed (5mm steel beads agitated for 20 seconds at 15 Hz, followed by 0.1mm glass beads for 5 minutes at 30 Hz). The same extraction procedure was carried out for the tissue biopsy samples. The remainder of the extraction protocol was performed as per the Qiagen DNeasy Blood & Tissue Kit instructions (Qiagen, CA, USA). Extracted DNA was stored at -80°c until sequencing.

### PCR amplification of 16S rRNA gene and pyrosequencing

Tag-encoded FLX-Titanium amplicon pyrosequencing for bacterial organisms was performed as previously described.[20] Briefly, a selective panbacterial 16S-rRNA gene-directed primer set (“27Fmod” AGRGTTTGATCMTGGCTCAG; and “519Rmodbio” GWATTACCGCGGCKGCTG) was applied against the 16S rRNA gene for PCR amplification. PCR and pyrosequencing was performed by MR DNA Lab (Shallowater, TX). A total of 28 cycles of PCR were performed. DNA was normalized at 7ng/μl average. Sequencing was performed on a Roche 454 sequencer (F. Hoffmann-La Roche Ltd, Basel, Switzerland). All samples included in this study were sequenced in one run.

### Bioinformatics pipeline

Raw pyrosequencing data files were then processed using the open-source software pipeline “Quantitative Insights into Microbial Ecology” (QIIME) version 1.8,[21] utilizing virtual machines on the Australian National eResearch Collaboration Tools and Resources (NeCTAR) research cloud. Sequences were trimmed of barcodes and primers and sequence quality control was performed using QIIME's default script settings (sequence length 200–1000 nucleotides; minimum average qualilty score 25; maximum length of homopolymer runs 6; maximum number of ambiguous bases 6). Sequences were then denoised using QIIME's built-in denoiser, set on the Titanium profile.[22] Operational taxonomic units (OTUs) were clustered (open-reference OTU “picking” against the August 2013 reference Greengenes 16S rRNA database[23]) at 97% similarity using *uclust*,[24] and then singleton sequences were removed. Taxonomic assignment of OTUs was then performed using BLAST[25] against the Greengenes 16S rRNA database.[23] After examining read counts, rarefactions of the OTU table were performed to a chosen maximum subsampling depth of 1100 sequences and rarefaction curves were plotted. Summary of taxonomic assignments were plotted as bar charts using QIIME. Observed species richness, as well as phylogenetic and non-phylogenetic alpha diversity metrics (Faith's Phylogenetic Diversity index “PD_Whole_Tree” and Shannon's index, respectively) were recorded and compared at the 1100 rarefaction depth. Phylogenetic and non-phylogenetic beta diversity matrices (Weighted/Unweighted UniFrac, and Bray-Curtis, respectively) were calculated. Three-dimensional Principal Coordinate Analysis (PCoA) plots were generated using EMPeror software[26] bundled with QIIME. Using the principal coordinates of the PCoA plots, a Procrustes transformation was performed (over the first three principal components) of the swab samples against those of the tissue samples. This was done using the QIIME script transform_coordinate_matrices.py. Using this script, an *m* ^*2*^ *value* is calculated, and Monte-Carlo simulations (1000 permutations) were done to calculate a p-value.

### Statistical analysis

All statistics were performed using R statistical software[27] (R Foundation for Statistical Computing, Vienna, Austria) through the IPython notebook interface.[28] Statistical significance was considered at the 0.05 level. Alpha diversity metrics were compared between the two sample types using Wilcoxon signed rank test. Beta diversity distances within-group were compared to between-group non-parametrically using a 999-permutations t-test. Testing for the presence of a significant effect of sample type on beta diversity metrics was also done using permutational multivariate analysis of variance[29] (PERMANOVA), through the function “adonis” present in the vegan R package.[30] To accommodate the paired design, the adonis function was employed using the strata parameter; this allowed the permutations to be done only within the patient variable, not across. We then investigated statistically significant differential relative abundance (MRA) of any bacterial species (of more than 1%) between the two groups using Wilcoxon signed rank tests. Correlation between the taxonomic assignment summaries of the two groups (Pearson's Correlation coefficient) was calculated non-parametrically using a two-sided 999 permutations test, using the QIIME script compare_taxa_summaries.py. This script (in paired mode) compares pairs of samples across two groups (within patient) and additionally calculates a summarized value for the whole comparison.

## Results

### Demographic and clinical data

In total, six CRS patients undergoing endoscopic sinus surgery were included in this study. Two of the patients had CRS without nasal polyposis (CRSsNP) and four had nasal polyposis (CRSwNP). Five patients had concomitant asthma. Two patients had aspirin sensitivity.

### Taxonomic summary

**Fig 1:**
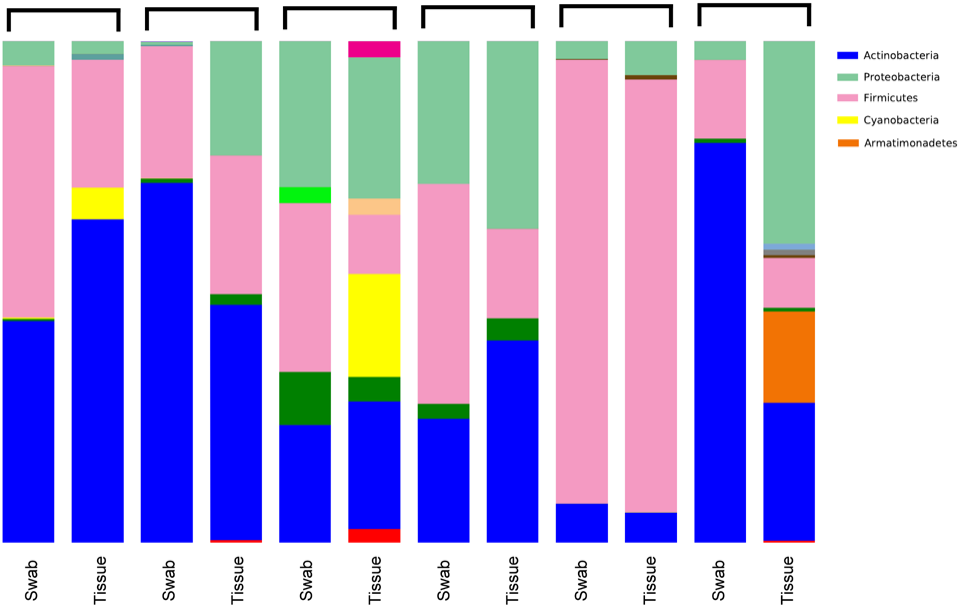
Distribution of bacterial phyla across all samples.

The mean number of sequences per sample was 2865.333 sequences (SD 2805.204). Our final OTU table contained 1169 unique OTUs (at 97% similarity) in 12 samples (6 tissues, 6 swabs). Upon taxonomy assignment, these OTUs represented 312 unique bacterial genera across 24 phyla. Fig 1 shows the distribution of bacterial phyla in the studied samples. Only 10 genera, out of the 312, had a mean relative abundance of more than 1%, and these are listed in Table 1.

**Table 1:** Most abundant taxa in our cohort.

Taxon	MRA (both groups' average)	MRA (swab)	MRA (tissue)	Difference in MRA	p-value^†^	FDR-corrected p-value^††^
g. Corynebacterium	35.00	47.80	22.20	25.6	>0.05	>0.05
g. Staphylococcus	30.20	34.60	25.80	8.8	0.031*	>0.05
g. Propionibacterium	3.80	2.80	4.70	1.9	>0.05	>0.05
g. Pseudonocardia	2.70	0.20	5.20	5	>0.05	>0.05
g. Anaerococcus	1.70	2.10	1.20	0.9	>0.05	>0.05
Family Comamonadaceae (genus not assigned)	1.60	1.10	2.10	1	>0.05	>0.05
Family Xanthomonadaceae (genus not assigned)	1.40	0.10	2.60	2.5	>0.05	>0.05
g. Peptoniphilus	1.40	1.60	1.10	0.5	>0.05	>0.05
g. Beijerinckia	1.10	0.00	2.30	2.3	>0.05	>0.05
Family Neisseriaceae (genus not assigned)	1.10	1.80	0.40	1.4	>0.05	>0.05

^†^ Wilcoxon signed rank test between tissue and swab groups

^††^ FDR = Benjamini-Hochberg’s False Discovery Rate

* = p-value less than 0.05

MRA = Mean Relative Abundance (%)

### Effect of sample type on observed species richness and alpha diversity

**Fig 2:**
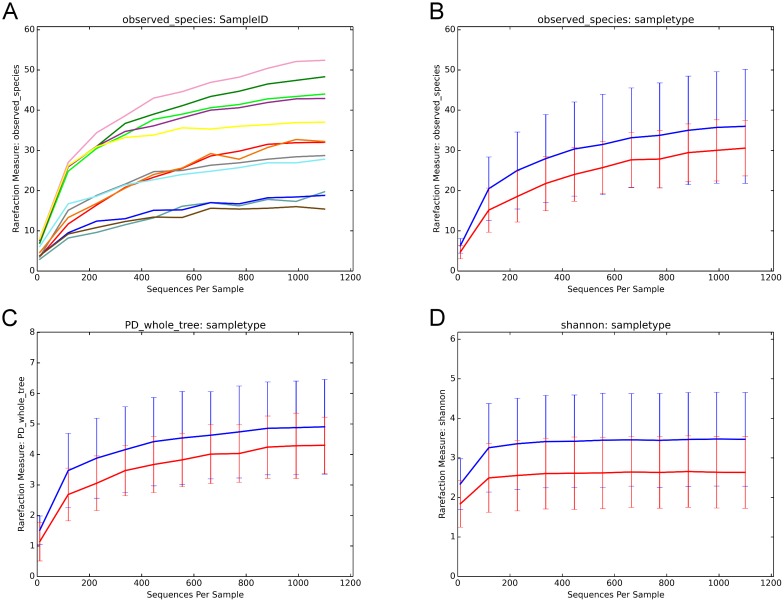
Rarefaction plots. (2A) Rarefaction plots of all 12 samples (one curve per sample) showing curves reaching asymptote at the cut-off of 1100 reads. (2B) Rarefaction curves (number of observed species on Y axis) for mucosal tissue (blue curve) versus swab (red curve). Richness at the 1100 cut-off was 36 for mucosal tissue, versus 30.6 for swab. (p > 0.05). (2C) Rarefaction curves (Faith’s Phylogenetic Diversity “PD_Whole_Tree” on Y axis) for mucosal tissue (blue curve) versus swab (red curve). (p > 0.05). (2D) Rarefaction curves (Shannon’s index on Y axis) for mucosal tissue (blue curve) versus swab (red curve).

Rarefactions were performed to a depth of 1100 reads. At the maximum subsampling depth of 1100 reads, the mean observed species richness was 33.3 species (36 species for mucosal tissue, versus 30.6 species for swab). Rarefaction curves for richness in all 12 samples plateau at the maximum depth, (Fig 2A) indicating an adequate sampling procedure. There was no significant difference in the observed species richness between both groups (Wilcoxon Signed Rank test, p = 0.44). There was no significant difference in the phylogenetic and non-phylogenetic alpha diversity metrics (Faith’s PD_Whole_Tree and Shannon’s index) between the two sampling methods (Wilcoxon Signed Rank test, p = 0.44 and p = 0.09, respectively).

### Effect of sample type on beta diversity

**Fig 3:**
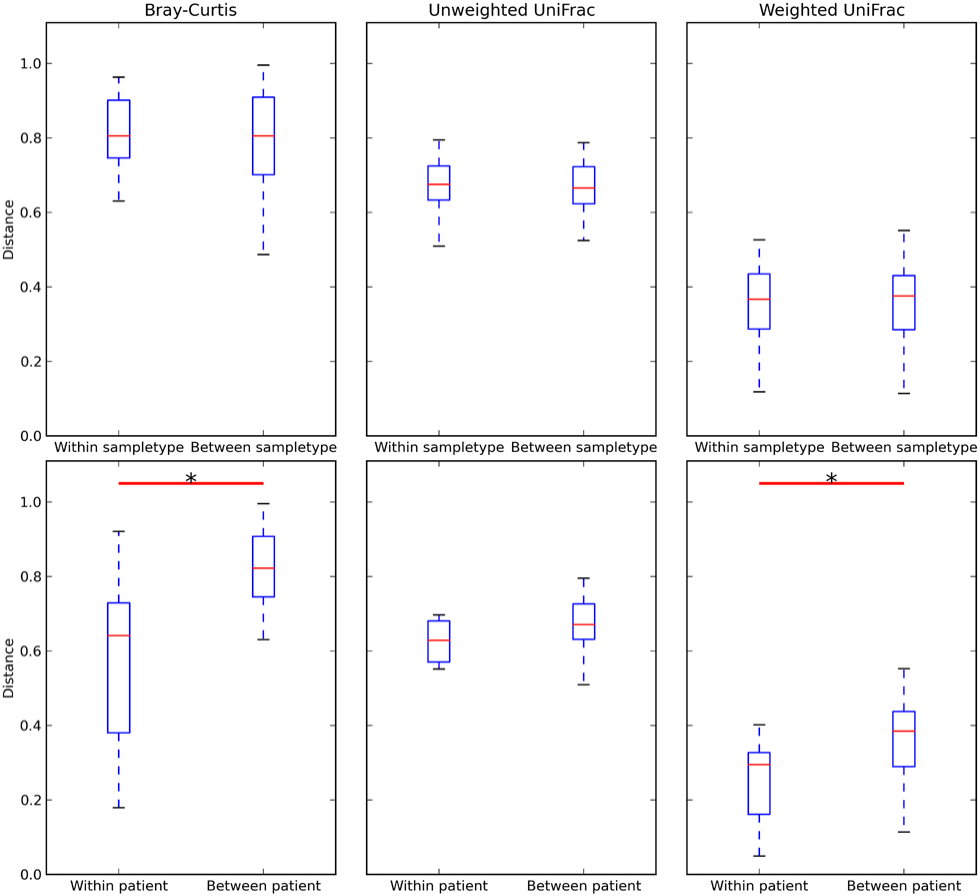
Boxplots showing distances from Bray-Curtis, unweighted UniFrac, and Weighted UniFrac distance matrices. Boxplots show no significant difference between within-sampletype and between-sampletype distances, and a statistically significant difference between within-patient and between-patient Bray-Curtis and Weighted UniFrac distances. Whiskers extend to cover the whole range of values. * = p < 0.05 (two-tailed Student's t-test, computed through QIIME’s script make_distance_boxplots.py).

Beta diversity distance matrices were generated, using Weighted/unweighted UniFrac and Bray-Curtis distances between all samples. The mean distances between samples within the same sample type group (i.e. within-swab, as well as within-tissue) did not differ significantly from mean distances between samples across sample type groups (i.e. between swab and mucosa). (Weighted UniFrac, unweighted UniFrac, Bray-Curtis; p > 0.05; Fig 3) This indicates that samples within each group were as similar to each other as to samples across the two groups. Moreover, the mean Bray-Curtis and Weighted UniFrac within-patient distances were significantly lower than between-patient distances (p < 0.05; Fig 3), indicating lower diversity and closer similarity within each patients paired samples (versus between-patient). Similarly, the within-patient unweighted UniFrac distances were also lower than the between-patient distances but this was not statistically significant. (Fig 3) We then examined the effect of sample type on the Weighted and unweighted UniFrac (phylogenetic) and Bray-Curtis (non-phylogenetic) distance matrices using PERMANOVA, while constraining permutations within the patient variable to account for the paired design. We found no evidence of significant impact of sample type on the Unweighted UniFrac distance matrix (pseudo-F-statistic = 0.93, p = 0.35), Weighted UniFrac distance matrix (pseudo-F-statistic = 1.03, p = 0.06), as well as on the Bray-Curtis distance matrix (PERMANOVA pseudo-F-statistic = 0.63, p = 0.41).

### Principal Coordinate Analysis (PCoA) and Procrustes Analysis

**Fig 4:**
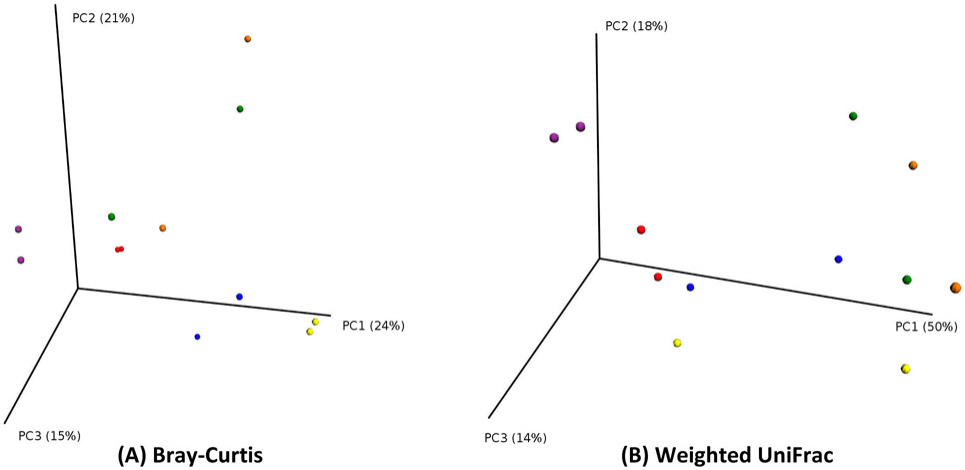
PCoA plots (by patient). PCoA plot showing good clustering of pairs of samples originating from the same patient. Points in three-dimensional space represent samples, each colored according to the patient. Each patient has two samples. (PCoA of Bray-Curtis distances on the left; PCoA of Weighted UniFrac distances on the right).

**Fig 5:**
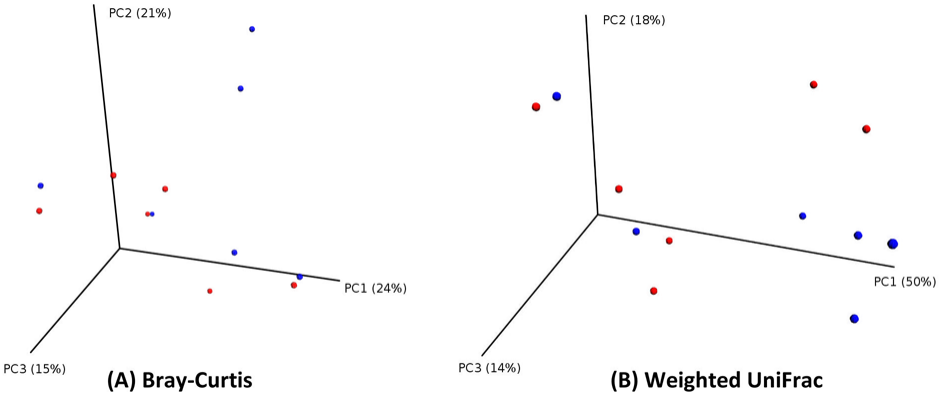
PCoA plots (by sample type). PCoA plots showing less defined clustering of samples, each colored according to the sample type (red for swab; blue for tissue). (PCoA of Bray-Curtis distances on the left; PCoA of Weighted UniFrac distances on the right).

We then examined beta diversity distances between samples using Principal Coordinate Analysis (PCoA). The PCoA plots show good clustering of the pair of samples collected from the same patient, but shows less defined clustering of swab versus tissue samples. (Figs 4 and 5).

**Fig 6:**
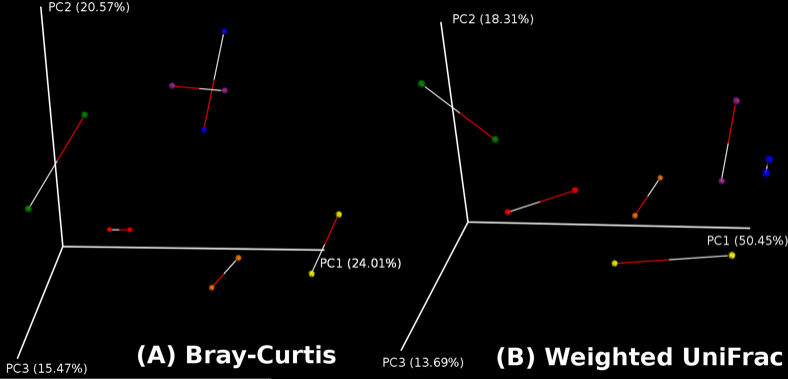
Procrustes plots. Using the principal coordinates of the PCoA plots, Procrustes transformation on (the first three) principal coordinates of the swab samples over those of the tissue samples was done.

Procrustes analysis was then done on these PCoA plots, such that to compare the principal coordinates of the swab samples to those of the tissue samples. The *m* ^*2*^ statistic produced by the Procrustes analysis is a value that can range from 0 (in this case the matrices are identical/highly similar) to 1 (matrices are completely dissimilar). The values obtained were: *m* ^*2*^ = 0.293, Monte-Carlo *p* = 0.020 (for Bray-Curtis PCoA) and *m* ^*2*^ = 0.368, Monte-Carlo *p* = 0.055 (for Weighted UniFrac PCoA). Procrustes plots can be found in Fig 6.

### Investigating significant differential abundance of species between both sampling methods

We then used Wilcoxon ranked sum tests to compare mean relative abundances (MRA) of bacterial genera in the two groups. We only found one statistically significant result (Staphylococci, 8.8% difference in MRA; 34.6% in swab versus 25.8% in tissue), however this did not remain significant after correction for multiple comparisons using the Benjamini-Hochberg False Discovery Rate (FDR). (Table 1) No other genera showed a statistically significant differential abundance between the two sampling methods. (p > 0.05) The largest difference in MRA between swab and tissue was found in Corynebacterium (higher abundance in swabs, 25.6% difference in MRA; Table 1)

### Pearson's correlation coefficient between the two sampling methods

Taxa summaries in both types of sample were compared in each patient in a paired method, using Pearson's correlation coefficient. This was calculated using the QIIME script compare_taxa_summaries.py. The summarized overall coefficient calculated by the script was 0.77 [95% CI:0.75 to 0.78] (p = 0.001), indicating a strong correlation of taxonomic assignment between both sampling methods. The detailed within patient paired correlation results are found in Table 2, which shows a strong correlation between all pairs except for one pair of samples which showed no or weak correlation.

**Table 2:** Pairwise, within-patient correlations as calculated by the QIIME script compare_taxa_summaries.py.

Sample pair	Pearson correlation coefficient	Non-parametric p-value (999 permutations)
1	0.8956	0.001
2	0.8446	0.001
3	0.0961	0.038
4	0.315	0.008
5	0.9818	0.001
6	0.7289	0.001

The table shows significant correlations in five out of the six sample pairs.

## Discussion

In this study, we investigated whether there was a significant difference in microbiome results between swab and mucosal tissue samples obtained from the same site of the same patients. We investigated this through alpha diversity, beta diversity, PCoA, as well as the taxonomy assignments. Our results suggest a good correlation between swabs and mucosal tissue samples, with no major significant differences in bacterial composition.

We found that mucosal tissue biopsy samples had higher observed species richness (30.6 species for swab, versus 36 species for mucosa) as well as alpha diversity indices (Faith’s and Shannon’s indices) (Fig 2B, 2C and 2D). However, this difference in richness and diversity did not reach statistical significance between the two groups. The plots in Fig 2A show that rarefaction curves have plateaued and reached asymptote. This indicates good sampling at the chosen cutoff depth of 1100 reads, and that a higher depth of sequencing is unlikely to discover more unique species (and increased diversity) for the sampled environment. We then explored whether the type of sample would have an effect on beta diversity metrics (Bray-Curtis and Weighted and unweighted UniFrac), and this showed no significant effect of sample type on beta diversity. (Fig 3) PCoA plots suggested a better clustering of samples by patient, rather than the type of sample. (Figs 4 and 5) Moreover, we calculated a high Pearson’s correlation coefficient between the taxa summaries of both sample types (r = 0.77, p = 0.001).

Previous studies showed considerable variability in sinus microbiota between individuals. We believe comparing the microbiota of tissue biopsy and swab samples obtained from the same patient controls for this inter-patient variability. Both sample types were taken from the same sites. This paired comparison design is one of the strengths of this study, as it increases its statistical power and minimizes the effect of confounding variables.

The alpha and beta diversity metrics (Faith’s PD_Whole_Tree, Shannon’s, Bray-Curtis, Weighted UniFrac) measured in this study were particularly chosen for the following reasons. First, these measures include both non-phylogenetic metrics (Shannon’s for alpha diversity and Bray-Curtis for beta diversity) as well as phylogenetic metrics (Faith’s PD_Whole_Tree for alpha diversity and UniFrac for beta diversity). Phylogenetic metrics include information about the evolutionary distance between taxa, since they account for the structure of the phylogenetic tree. Non-phylogenetic metrics do not account for this information and only depend on taxa abundance (or prevalence) and evenness. Second, we have also included metrics that account for the relative abundance of taxa (Bray-Curtis, Weighted UniFrac), contrary to using only metrics such as Jaccard’s or unweighted UniFrac that depend on absence/presence (i.e. prevalence) of taxa, without accounting for their relative abundance. In this way, we could demonstrate that both methods of sampling (tissue and swab) are not only similar in regards to absence/presence of taxa, but also in regards to their relative abundance within each sample. Despite mucosal tissue samples having higher richness and within-community (α) diversity, we could not demonstrate that this was significantly higher than swab samples.

Some analyses in our study approached statistical significance (for example, Shannon’s index comparison p value was 0.09 and Weighted UniFrac Permanova p = 0.06), which may imply the presence of a significant difference between swab and tissue samples, especially given the small number of samples in our cohort. Nevertheless, we believe positive results of all analysis types in the current study, which includes other metrics (alpha diversity metrics, beta diversity distances, PCoA plots, Pearson’s correlation), supports a general conclusion of tissue and swab similarity. This raises the question of power in the realm of microbiota comparison studies, which is a subject of ongoing research.[31,32] Moreover, what is the *clinically*-significant “effect size” calculated out of the commonly used diversity measures, and that would correlate with sinus health and/or disease? These questions need to be better defined for future research. Future studies confirming the findings of our study may benefit from including a larger cohort. Another caveat with our current study design is the possibility that taking the swab samples (first) somehow perturbed the surface microbiome of the tissue, which was biopsied from the same site after swabbing (middle meatus/anterior ethmoid region). Although this limitation seems unavoidable with the current study design, we believe this issue would not cause a significant change to the tissue specimens, since the tissue biopsied is typically larger than the surface area covered by the swab head, and thus is not limited only to the thin strip touched by the swab.

Although our rarefaction curves indicate a saturation of diversity at our chosen cut-off rarefaction depth (Fig 2C and 2D), future studies should investigate using the Illumina sequencing platform, which allows sequencing at ultra-high depths, with millions of reads generated per run.[33] The Illumina platform is thus better poised to examine the population belonging to the “rare biosphere”, and thus the only limitation to accurately characterizing this rare population would be sequencing errors or limitations of the bioinformatics analysis such as imperfect OTU clustering.[34,35] In our study, it appears that the UniFrac metric (which does not take abundance of the taxa into account, giving abundant and rare taxa equal weight) is less able to show the within-patient similarity of swab and tissue samples, in contrast to Bray-Curtis or Weighted UniFrac (Fig 3). This may suggest either a less than perfect ability of swab samples to accurately characterize the rare biosphere, or is an artefact of the OTU clustering. This is an additional reason to confirm the findings of this experiment on the Illumina platform in the future. Some sinonasal microbiome studies also used lavage sampling.[2,6] Unfortunately, this type of sampling was not investigated in our study.

Although the aim of this study is not to describe the CRS sinonasal microbiome, we also report on the taxa discovered (Table 1). Our findings show that *Corynebacteria*, *Staphylococci* and *Propionibacteria* are the most abundant micro-organisms. (Table 1) These genera were also reported in previous studies as abundant in the sinuses.[4,36]. Interestingly, out of the 312 bacterial genera assigned in our study, only 10 (about 3%) had a mean relative abundance of more than 1% (Table 1). This suggests the presence of a “rare biosphere” in the sinuses. This rare biosphere may constitute then about 97% of all species present in the sinuses. However the role played by these rare taxa, and whether they contribute significantly to sinus health or disease, is still unknown; although other studies suggested they have great functional importance at other sites such as the gut and oral microbiota.[37,38] With this said, the small sample size and lack of a healthy control group makes it difficult to draw further conclusions on the sinus microbiome in CRS patients.

## Conclusion

In summary, our results suggest that there is no significant difference between mucosal tissue and swab samples and both methods showed strong correlation. We therefore propose that swab samples are sufficiently representative of the sinonasal mucosa microbiome and therefore can be used for future sinonasal microbiome studies. This obviates the need for invasive mucosal biopsies and also means that sinus microbiome swabs can be obtained from healthy and diseased patients intra-operatively, as well as post-operatively in the clinic. Future studies confirming these findings should explore: investigating sinus lavage samples, including a higher number of individuals, and using sequencing platforms that allow ultra-high depths of sequencing.

## Supporting Information

S1 File QIIME taxa plots at the level of genus. (ZIP)
