# Supplementary figures and images for: Sinonasal Microbiome Sampling: A Comparison of Techniques
Source: PLoS One. 2015 Apr 14;10(4):e0123216. doi: 10.1371/journal.pone.0123216 (PMC4396979; doi:10.1371/journal.pone.0123216)

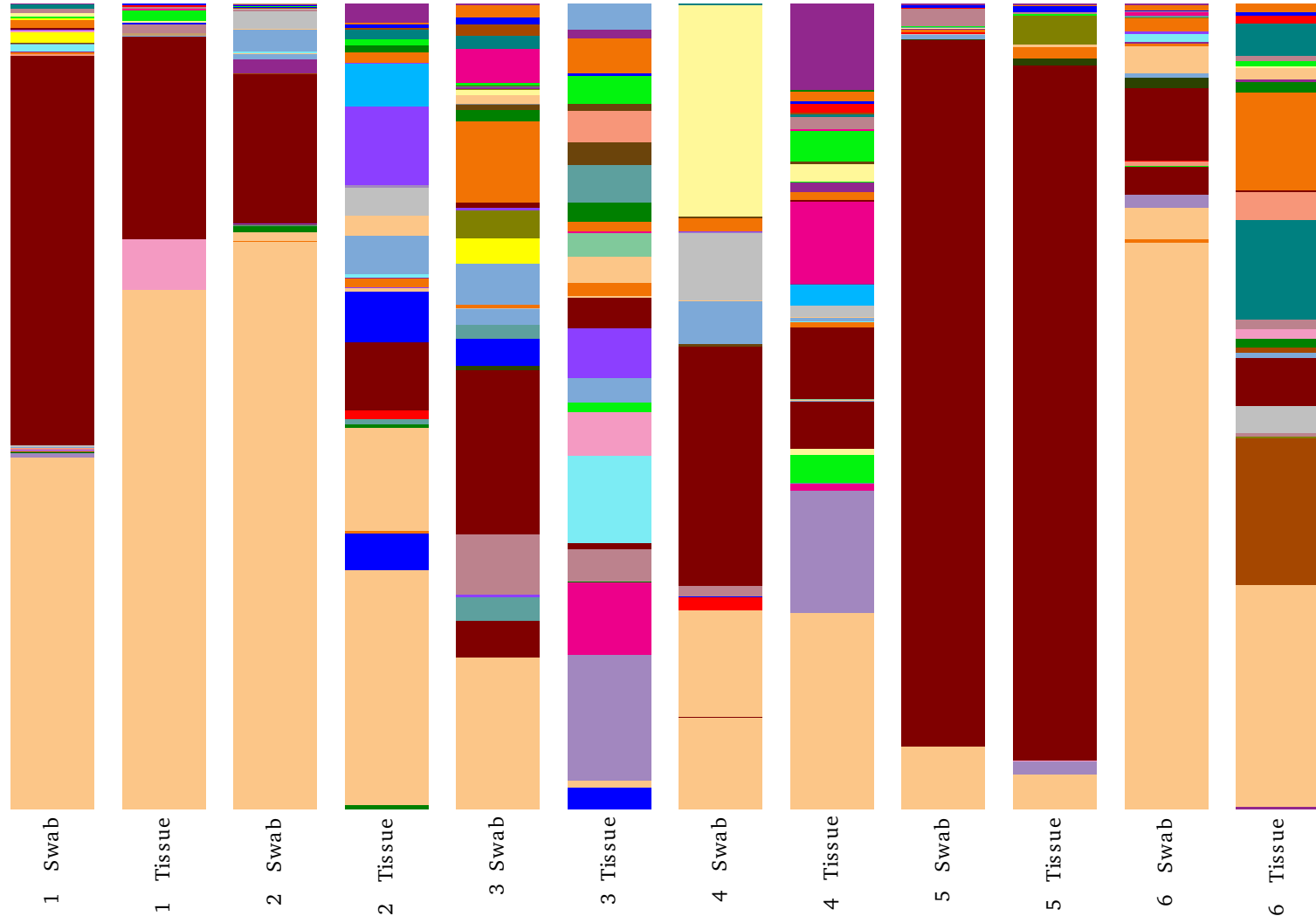

Supplement: S1 File — (ZIP) [file pone.0123216.s001.zip › taxa_plots_genera.pdf]
